# Persistence of immune responses to the Sinopharm/BBIBP‐CorV vaccine

**DOI:** 10.1002/iid3.621

**Published:** 2022-05-11

**Authors:** Chandima Jeewandara, Inoka Sepali Aberathna, Pradeep Darshana Pushpakumara, Achala Kamaladasa, Dinuka Guruge, Ayesha Wijesinghe, Banuri Gunasekera, Shyrar Tanussiya Ramu, Heshan Kuruppu, Thushali Ranasinghe, Shashika Dayarathna, Osanda Dissanayake, Nayanathara Gamalath, Dinithi Ekanayake, Jeewantha Jayamali, Deshni Jayathilaka, Madushika Dissanayake, Tibutius Thanesh Jayadas, Anushika Mudunkotuwa, Gayasha Somathilake, Michael Harvie, Thashmi Nimasha, Saubhagya Danasekara, Ruwan Wijayamuni, Lisa Schimanski, Pramila Rijal, Tiong K. Tan, Tao Dong, Alain Townsend, Graham S. Ogg, Gathsaurie Neelika Malavige

**Affiliations:** ^1^ Allergy Immunology and Cell Biology Unit, Department of Immunology and Molecular Medicine University of Sri Jayewardenepura Nugegoda Sri Lanka; ^2^ Public Health Department Colombo Municipal Council Colombo Sri Lanka; ^3^ MRC Human Immunology Unit, MRC Weatherall Institute of Molecular Medicine University of Oxford Oxford UK; ^4^ Centre for Translational Immunology, Chinese Academy of Medical Sciences Oxford Institute University of Oxford Oxford UK

**Keywords:** antibody, COVID‐19, kinetics, persistence, Sinopharm/BBIBP‐CorV, vaccines

## Abstract

**Background:**

To determine the kinetics and persistence of immune responses following the Sinopharm/BBIBP‐CorV, we investigated immune responses in a cohort of Sri Lankan individuals.

**Methods:**

SARS‐CoV‐2 specific total antibodies were measured in 20–39 years (*n* = 61), 40–59 years (*n* = 120) and those >60 years of age (*n* = 22) by enzyme‐linked immunosorbent assay, 12 weeks after the second dose of the vaccine. Angiotensin‐converting enzyme 2 (ACE2) receptor blocking antibodies (ACE2R‐Ab), antibodies to the receptor‐binding domain (RBD) of the ancestral virus (WT) and variants of concern, were measured in a sub cohort. T cell responses and memory B cell responses were assessed by ELISpot assays.

**Results:**

A total of 193/203 (95.07%) of individuals had detectable SARS‐CoV‐2 specific total antibodies, while 67/110 (60.9%) had ACE2R‐Ab. A total of 14.3%–16.7% individuals in the 20–39 age groups had detectable antibodies to the RBD of the WT and variants of concern, while the positivity rates of those ≥60 years of age was <10%. A total of 14/49 (28.6%) had Interferon gamma ELISpot responses to overlapping peptides of the spike protein, while memory B cell responses were detected in 9/20 to the S1 recombinant protein. The total antibody levels and ACE2R‐Ab declined from 2 to 12 weeks from the second dose, while ex vivo T cell responses remained unchanged. The decline in ACE2R‐Ab levels was significant among the 40–59 (*p* = .0007) and ≥60 (*p* = .005) age groups.

**Conclusions:**

Antibody responses declined in all age groups, especially in those ≥60 years, while T cell responses persisted. The effect of waning of immunity on hospitalization and severe disease should be assessed by long term efficacy studies.

## Graphical abstract

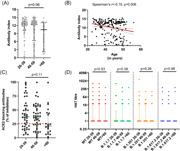

## 1 INTRODUCTION

The Sinopharm/BBIBP‐CorV is an inactivated COVID‐19 vaccine, which is currently approved in 65 countries^1^ and it has been the most widely used vaccine in Sri Lanka. The phase 3 clinical trials showed an efficacy of 78.1% against symptomatic illness,^2^ while all individuals were reported to have seroconverted and shown to have developed neutralizing antibodies by 42 days, following the second dose of the vaccine.^3^ We previously reported that 95% of Sri Lankan individuals seroconverted 2 weeks following the second dose, with lower seroconversion rates in older individuals.^4^ Angiotensin‐converting enzyme 2 (ACE2) receptor blocking antibodies, measured by the surrogate virus neutralizing test (sVNT) were found in 81.25% of individuals following both doses of the vaccine, while antibodies to the receptor‐binding domain (RBD) was significantly less than those following natural infection.^4^ Although Sinopharm/BBIBP‐CorV is the main vaccine used by many Asian and Middle East countries, there are limited data regarding its efficacy when used in different countries and for different variants. Furthermore, data regarding the persistence of antibody and T cell responses in fully vaccinated individuals are limited.

Neutralizing antibodies and non‐neutralizing antibodies specific to the spike protein have shown to gradually decline following the second dose of BNT162b2 (Pfizer–BioNTech) and AZD1222,^5^ while the messenger RNA (mRNA)‐1273 vaccine was shown to induce stable responses up to 6 months after the second dose.^6^ Although the quantity of neutralizing antibodies that are needed for protection from severe disease or to prevent breakthrough infection is not known, it has been shown that the efficacy of BNT162b2 (Pfizer–BioNTech) vaccine declines with time.^7^ Booster doses of BNT162b2 (Pfizer–BioNTech) vaccine resulted in a 11.3‐fold reduction in acquisition of infection and 19.5‐fold reduction in severe disease compared those who did not receive a booster.^8^ However, the World Health Organization recently stated that although there is evidence of declining vaccine efficacy against mild illness, the efficacy of vaccines against hospitalization and severe disease appears to be high and therefore, more emphasis should be placed on vaccinating vulnerable individuals.^9^ Despite the decline in neutralizing antibodies following vaccination and natural infection with time, spike protein specific memory B cells have shown to persist for a longer duration.^10^ Although these long‐lived memory cells are thought to give long lasting protection against severe illness, fully vaccinated elderly individuals in Israel appear to have been susceptible to severe disease and thus benefited from a booster dose.^11^ Based on waning of neutralizing antibody responses and efficacy for certain COVID‐19 vaccines with time, booster doses are currently been offered to older individuals and individuals in high risk categories in different countries.^12^, ^13^

Although there are data regarding the duration and persistence of antibody and T cell responses following many COVID‐19 vaccines such as BNT162b2 (Pfizer–BioNTech), AZD1222 and mRNA‐1273^14^, ^15^ there are limited data regarding the persistence of immune responses following Sinopharm/BBIBP‐CorV.^16^ It is crucial that severe disease and hospitalizations due to COVID‐19 are curtailed to a level that it no longer becomes a global threat. While vaccinating all individuals worldwide is essential to end the pandemic, it is also important to monitor the duration of immunity and any changes in vaccine efficacy over time. Therefore, we sought to investigate the persistence of antibody and T cell responses in a Sri Lankan cohort to understand the durability of immune responses to this vaccine in different age groups.

## 2 METHODS

We previously evaluated immune responses to Sinopharm/BBIBP‐CorV in 323 Sri Lankan individuals from Colombo at 4 weeks after the first dose and 2 weeks after the second dose (6 weeks after the first dose).^4^ To determine the persistence of antibody and T cell responses following the second dose, blood samples were obtained at 3 months (12 weeks) following the second dose of the vaccine in 203 individuals from this cohort, while those who reported as being PCR positive/diagnosed of COVID‐19 or reported symptoms suggestive of COVID‐19 such as fever, sore throat, cough and myalgia were excluded from the 3‐month analysis. We also excluded individuals in whom a household member also had a diagnosed COVID infection, or if the individual showed symptoms suggestive of COVID‐19 during this time (fever, cough, sore throat). Demographic and the presence of comorbidities such as diabetes, hypertension, cardiovascular disease and chronic kidney disease was determined by a self‐administered questionnaire at the time of recruitment from all participants. Ethics approval was obtained from the Ethics Review Committee of University of Sri Jayewardenepura.

### 2.1 Detection of SARS‐CoV‐2 specific total antibodies, ACE2 receptor blocking antibodies, and antibodies to the RBD of SARS‐CoV‐2 VOCs

The Wantai SARS‐CoV‐2 Ab ELISA (Beijing Wantai Biological Pharmacy Enterprise) was used to detect the presence of SARS‐COV‐2 specific total antibodies (immunoglobulin [Ig]M, IgA, and IgG), which detects antibodies to the RBD of the spike protein, according to the manufacturer's instructions. The sVNT was used to measure ACE2‐receptor blocking antibodies and the haemagglutination test (HAT) was used to measure antibodies to the RBD of VOCs in a subcohort of individuals (*n* = 110). The Svnt was carried out as previously described according to the manufacturer's instructions (Genscript Biotech).^17^ This measures the percentage of inhibition of binding of the RBD to recombinant ACE2 and an inhibition percentage ≥25% in a sample was considered as positive for ACE2 receptor blocking antibodies.^18^

The HAT was carried out using the WT, B.1.1.7 (N501Y), B.1.351 (N501Y, E484K, K417N) and B.1.617.2 versions of the IH4‐RBD reagents,^19^ which included the relevant amino acid changes introduced by site directed mutagenesis. The assays were carried out and interpreted as previously described and a titre of 1:20 was considered as a positive response.^20^, ^21^ The HAT titration was performed using 7 doubling dilutions of serum from 1:20 to 1:1280, to determine presence of RBD‐specific antibodies. The RBD‐specific antibody titre for the serum sample was defined by the last well in which the complete absence of “teardrop” formation was observed.

### 2.2 Ex vivo interferon gamma (IFNγ) ELISpot assays and B cell ELISpot assays

Ex vivo IFNγ ELISpot assays were carried out using freshly isolated peripheral blood mononuclear cells (PBMCs) obtained from 49 individuals in whom we had previously carried out these assays at 4 and 6 weeks. Ex vivo IFNγ ELISpot assays were carried out using freshly isolated PBMCs. Two pools of overlapping peptides named S1 (peptide 1–130) and S2 (peptide 131–253) covering the whole spike protein (253 overlapping peptides) were added at a final concentration of 10 µM and incubated overnight.^22^, ^23^ All experiments were done in duplicate and phytohemaglutinin (PHA) was included as a positive control while media alone was used as a negative control. Briefly, ELISpot plates (Millipore Corp.) coated with anti‐human IFNγ monoclonal capture antibody overnight (Mabtech), were incubated overnight at 37°C and 5% CO_2_ at a concentration of 100,000 cells/well. The plates developed with a second biotinylated antibody to human IFNγ and subsequently developed with streptavidin‐alkaline phosphatase (Mabtech AB) and colorimetric substrate. The spots were enumerated using an automated ELISpot reader (AID Germany). Background (PBMCs plus media alone) was subtracted and data expressed as number of spot‐forming units (SFU) per 10^6^ PBMCs. A positive response was defined as mean ± 2 *SD* of the background responses.

### 2.3 B cell ELISpot assays

Due to the limitations in the availability of PBMCs to carry out these assays, B cell ELISpots were only done in 28 individuals, in whom ex vivo ELISpot assays were carried out. Briefly, freshly isolated PBMCs were stimulated in a 24 well plate using IL‐2 and R848 (a TLR 7/8 agonist) in RPMI supplemented with 10% fetal bovine serum, 1% penicillin streptomycin and 1% glutamine at 4 million cells/well and incubated at 37°C with 5% CO_2_ for 3 days. They were then washed and rested overnight and 100,000 cells/well were added. A total of 50,000 cells/well were added to the positive control wells. A Human IgG ELISpot kit (Mabtech 3850‐2A) was used according to the manufacturer's instructions to quantify IgG‐secreting cells specific to SARS‐COV2 S1, S2 and N recombinant proteins, which were coated at 2 µg/ml in phosphate buffered saline. All experiments were carried out in duplicate and anti‐human IgG monoclonal capture antibodies, was used as a positive control, and media alone as a negative control. A positive response was defined as mean ± 2 *SD* of the background responses. The spots were enumerated using an automated ELISpot reader (AID Germany).

### 2.4 Statistical analysis

The 95% confidence intervals for seropositivity for each age category were calculated using the R software (version 4.0.3) and R‐studio (version 1.4.1106). Nonparametric tests such as Mann–Whitney and Kruskal–Wallis were performed at a confidence level of 95% to identify the statistically significant relationships between the age categories and the sex of the individuals with seropositivity and the levels of antibodies. Spearman's correlation coefficient was used to determine the correlation between antibody, T cell responses and the age of an individual. Moreover, Friedman Tests were performed to identify if there is any significant differences between the antibody levels of the participants with the four different sample collection time points. Similar analyses were carried out for sVNT and HAT results to check if there are any statistically significant differences among the time points.

## 3 RESULTS

### 3.1 SARS‐CoV‐2 total antibody responses in different age groups

**Figure 1:**
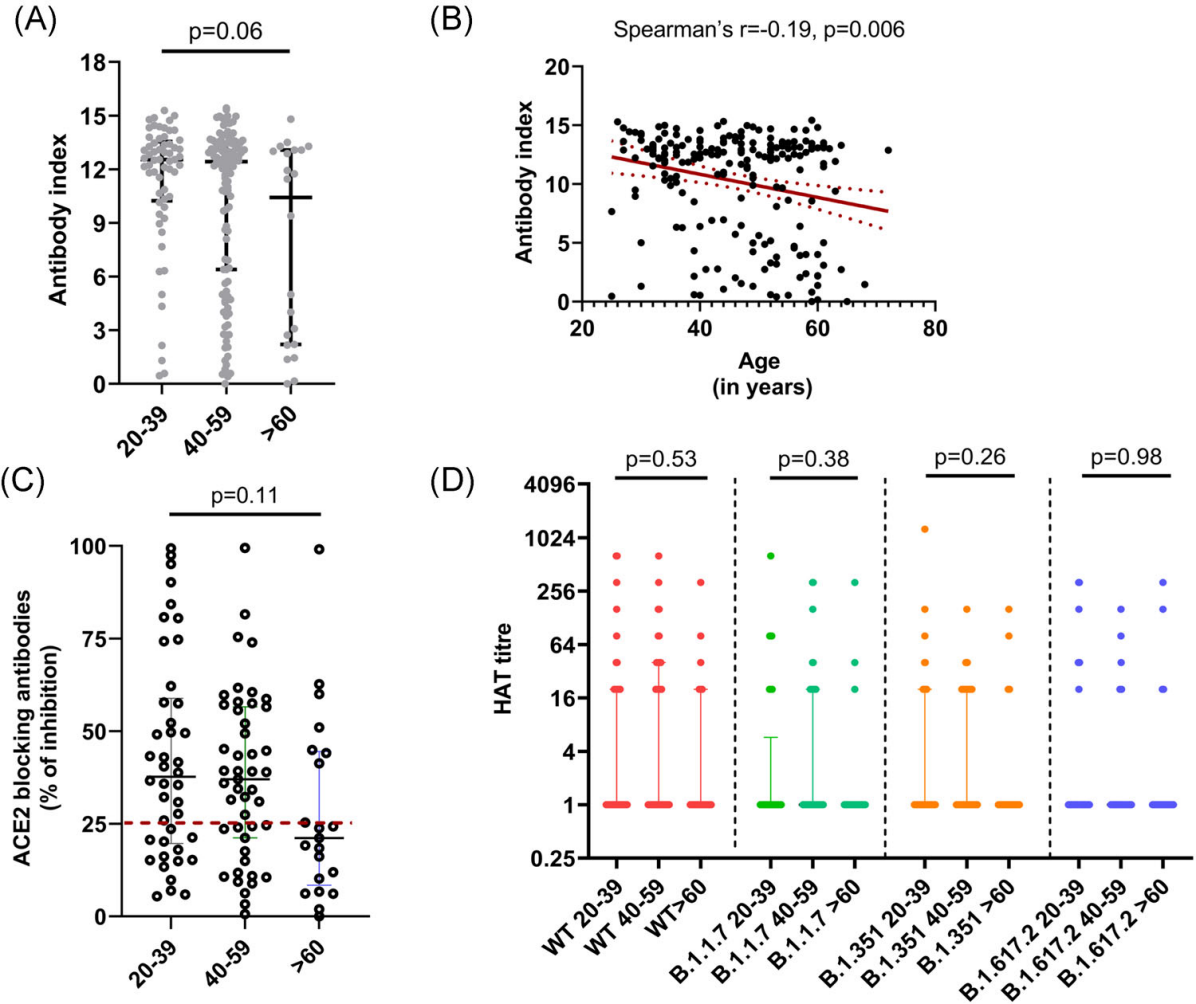
SARS‐CoV‐2 specific antibody responses 3 months following the second dose (16 weeks following the first dose) of the Sinopharm/BBIBP‐CorV vaccine. SARS‐CoV‐2 specific total antibodies were measured in 20–39 years old (*n* = 61), 40–59 years old (*n* = 120) and those ≥60 years of age (*n* = 22) by ELISA and no significant difference was seen between the age groups (*p* = .06) based on the Kruskal–Wallis test (A). The total antibody titres inversely correlated (Spearman's *r* = −.19, *p* = .006) with age (B) (the red dotted lines indicate the 95% confidence interval). The ACE2 receptor blocking antibodies were measured by the surrogate virus neutralizing test in 20–39 years old (*n* = 41), 40–59 years old (*n* = 48) and ≥60 years old (*n* = 21) and no significant difference was seen (*p* = .11) between the age groups based on the Kruskal–Wallis test (C). Antibodies were measured to the receptor‐binding domain of the ancestral SARS‐CoV‐2 virus (WT) and to B.1.1.7, B.1.351 and B.1.617.2 using the haemagglutination test (HAT) in 20–39 years old (*n* = 41), 40–59 years old (*n* = 48) and ≥60 years old (*n* = 21) and no significant difference was seen (*p* = .11) between the age groups based on the Kruskal–Wallis test for different variants (D). All tests were two‐tailed. The lines indicate the median and the interquartile range. ELISA, enzyme‐linked  immunosorbent assay

At 3 months (12 weeks) since receiving the 2nd dose 193/203 (95.07%) of individuals had detectable SARS‐CoV‐2 specific total antibodies. A total of 59/61 (96.72%) between the ages of 20−39, 114/120 (95.0%) of those 40–59 and 20/22 (90.91%) of those ≥60 years were seropositive. There was no significant difference (*p* = .06) between the antibody titres (indicated by antibody index) in the three different age groups (20–39, 40–59, and ≥60 years) (Figure 1A). However, the total antibody levels (indicated by the antibody index) significantly and inversely correlated with age (Spearman's *r* = −0.19, *p* = .006) (Figure 1B). There was no significant difference (*p* = .076) in the seropositivity rates or the antibody levels (indicated by antibody indices) in those with comorbidities, compared to those who did not have comorbidities. Similarly, the seropositivity rates between males and females were also not found to have a statistically significant difference (*p* = .3319).

### 3.2 SARS‐CoV‐2 specific ACE2‐receptor blocking antibodies in different age groups

The sVNT that measures ACE2 receptor blocking antibodies was carried out in a subset of individuals of cohort (*n* = 110) and 67 (60.9%) gave a positive result. A total of 27/41 (65.85%) in the 20–39 age group, 32/48 (66.67%) in the 40–59 age group and 8/21 (38.10%) of the ≥60 years age group gave a positive result. There was no significant difference (*p* = .06) between the ACE2 receptor blocking antibody positivity (% of inhibition ≥25) in different age groups. There were also no significant differences in the levels of ACE2 receptor blocking antibodies in different age groups (*p* = .11) (Figure 1C). However, there was a significant correlation (Spearman's *r* = .52, *p* < .0001) between SARS‐CoV‐2 specific total antibodies and levels of ACE2 receptor blocking antibodies.

### 3.3 Hemagglutination test (HAT) to detect antibodies to the RBD of SARS‐CoV‐2 and its variants of concern (VOCs)

The HAT assay was carried out to measure positivity rates and the antibody titres to the ancestral strain (WT), and the VOCs B.1.1.7, B.1.351 and B.1.617.2 in the same individuals in whom the sVNT assays were carried out (*n* = 110). The proportion of individuals who gave a positive result for the WT and the different VOCs and their mean, standard deviation, median values with the interquartile range (IQR) (HAT titres) are shown in Table 1. As determined by the Friedman test, the HAT titres for the WT were found to be significantly higher than the HAT titres to the different VOCs for the age groups 20–39 (*p* = .002) and 40–59 (*p* = .0001). The post hoc tests for multiple comparisons in the 20–39 group showed that the HAT titres for the WT was significantly higher than that for B.1.17 (*p* = .004) and B.617.2 (*p* = .002). In the 40–59 age group, the HAT titres to the WT were significantly higher than for B.1.1.7 (*p* = .001), B.1.315 (*p* = .006) and B.1.617.2 (*p* ≤ .001). However, the ≥60 years age group had low HAT titres for WT and all VOC, and there were no significant differences (*p* = .286) between responses (Figure 1D).

**Table 1:** Antibody responses to the receptor‐binding domain of the ancestral SARS‐CoV‐2 virus (WT) and to B.1.1.7, B.1.351, and B.1.617.2 using the haemagglutination test (HAT)

Age groups	WT	B.1.1.7 (alpha)	B.1.351 (beta)	B.1.617.2 (delta)
20–39 (*n* = 41)				
Numbr positive (%)	7 (16.7)	5 (11.9)	5 (11.9)	6 (14.3)
HAT titre mean (±*SD*)	50.7 (146.2)	25.9 (101.2)	42.0 (200.2)	22.9 (73.2)
HAT titre (IQR)	0 (20)	0 (0)	0 (20)	0 (0)
40–59 (*n* = 48)				
Number positive (%)	12 (25.5)	5 (10.6)	5 (10.6)	5 (10.6)
HAT titre mean (±*SD*)	38.8 (104.9)	22.5 (67.4)	12.5 (25.3)	8.9 (27.0)
HAT titre median (IQR)	0 (25)	0 (20)	0 (20)	0 (0)
≥60 (*n* = 21)				
Number positive (%)	3 (14.3)	2 (9.5)	2 (9.5)	2 (9.5)
HAT titre mean (±*SD*)	23.8 (70.6)	18.1 (69.8)	13.3 (38.1)	24.8 (76.1)
HAT titre median (IQR)	0 (20)	0 (0)	0 (0)	0 (0)

Abbreviation: IQR, interquartile range.

### 3.4 Ex vivo ELISpot responses in the different age groups

**Figure 2:**
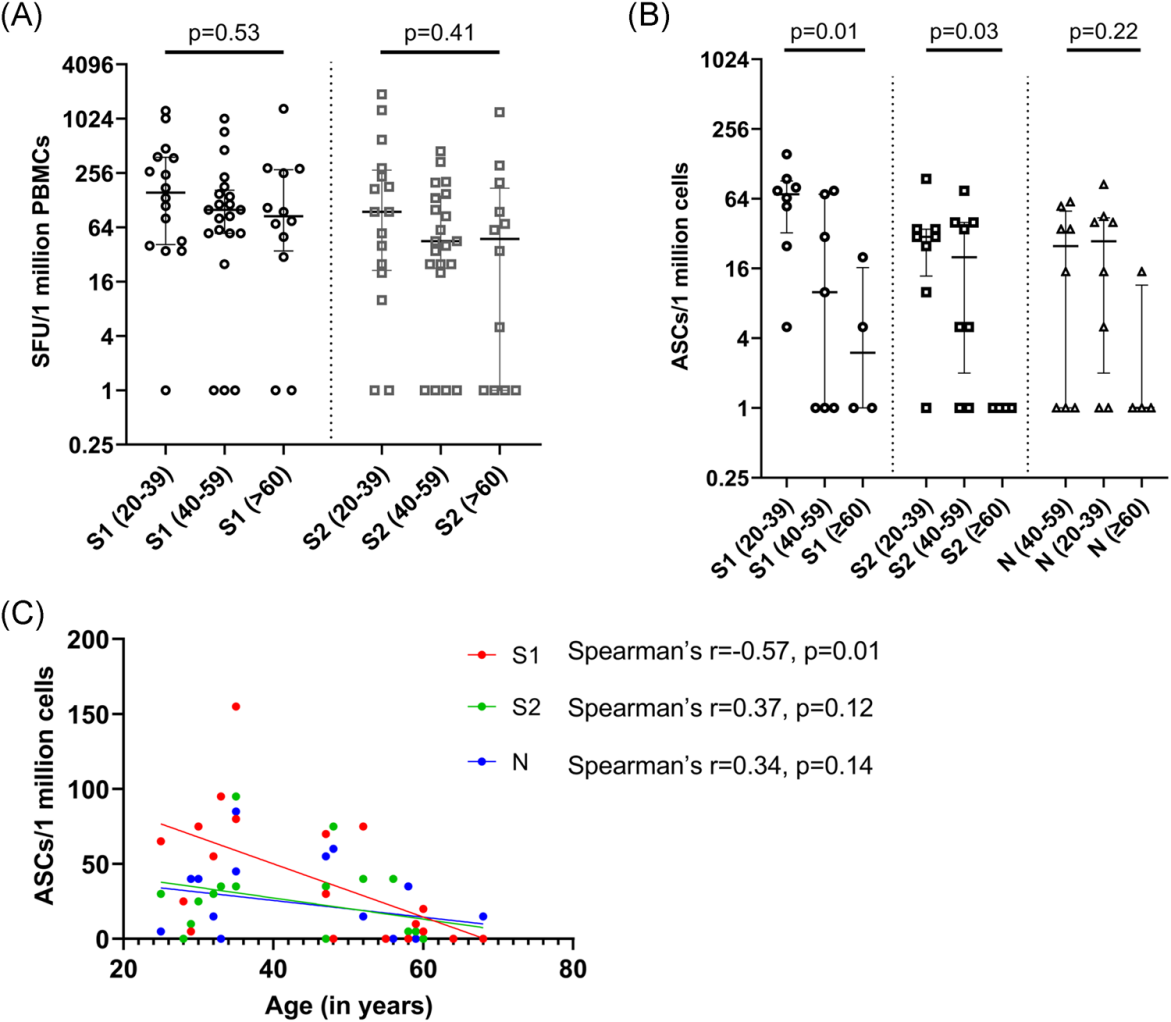
Ex vivo IFNγ ELISpots responses and B cell ELISpot responses 3 months following the second dose (16 weeks following the first dose) of the Sinopharm/BBIBP‐CorV vaccine. Ex vivo IFNγ ELISpots responses were measured to the S1 and S2 overlapping pool of peptides in 49 individuals between the age groups of 20–39 (*n* = 16), 40–59 (*n* = 21) and in ≥60 years olds (*n* = 12). There was no significant difference in the ex vivo ELISpot responses, for both S1 (*p* = .53) and S2 (*p* = .41) between the different age groups based on the Kruskal–Wallis test (A). The frequency of antibody secreting cells (ASCs) to S1, S2 and N recombinant proteins were assessed by B cell ELISpot assays in 20–39 (*n* = 8), 40–59 (*n* = 8), and in ≥60 years olds (*n* = 4). Significant differences in the responses to S1 (*p* = .01) and S2 (*p* = .03), but no difference to N (*p* = .22) in different age groups based on the Kruskal–Wallis test. The frequency of ASCs to the S1 protein significantly decreased with age (Spearman's *r* = −.57, *p* = .01), but not for S2 (Spearman's *r* = .37, *p* = .12) or N (Spearman's *r* = .34, *p* = .14) (C). IFNγ, interferon gamma

To investigate the T cell responses in these two cohorts, we carried out ex vivo IFNγ ELISpot responses in 49 individuals in different age groups, in those who were recruited by us to study the T cell responses. IFNγ ELISpot responses to S1 overlapping pool of peptides (median: 100, IQR: 47.5–260 SFU/1 million PBMCs) were significantly higher (*p* = .008) than those for the S2 pool (median: 55, IQR: 14–190 SFU/1 million PBMCs). The threshold for a positive response was set at 234 SFU/1 million PBMCs and accordingly, 14/49 (28.6%) of the individuals gave a positive response for S1. There was no significant difference in the ex vivo ELISpot responses for both S1 (*p* = .53) and S2 (*p* = .41) overlapping pool of peptides between the different age groups (Figure 2A). 7/16 (43.6%) of those between 20 and 39 years of age, 3/21 (14.3%) of those between 40 and 59 years of age and 4/12 (33.3%) of those ≥60 years old had a positive response to the S1 pool of peptides. After 16 weeks 9/49 (18.4%) individuals gave positive responses to the S2 peptide pool (Figure 2A). A total of 5/16 (31.3%) of those between 20 and 39 years of age, 2/21 (9.5%) of those between 40 and 59 years of age and 2/12 (16.7%) ≥60 years old had a positive response to S2 pool of peptides.

There was no correlation between the age of the individual and ex vivo IFNγ ELISpot responses to either S1 (Spearman's *r* = −.22, *p* = .12) or S2 (Spearman's *r* = −.19, *p* = .18) pool of peptides. The ex vivo overall IFNγ ELISpot responses significantly correlated with the SARS‐CoV‐2 specific total antibodies (Spearman's *r* = .33, *p* = .02), but not with ACE2 receptor blocking antibodies (Spearman's *r* = .20, *p* = .16).

### 3.5 The frequency of antibody secreting cells (ASCs) in those who received Sinopharm/BBIBP‐CorV

B cell ELISpot assays for S1, S2, and N recombinant proteins were carried out in 20/49 individuals, who were also included for evaluating of ex vivo T cell responses. The threshold of a positive response was set at 44.1 ASCs/1 million cells for S1, 32.9 for S2 and 19.1 for the N protein. 9/20 (45%) individuals gave a positive response for S1, 7/20 (35%) individuals responded to S2 and, 8/20 (40%) for the N protein. There was significant difference in the frequency of ASC to both S1 (*p* = .01) and S2 (*p* = .03) proteins between different age groups (Figure 2B). Individuals in the 20–49 age group showed higher frequency of ASCs to both S1 and S2 (S1—median: 70, IQR: 32–91 ASCs/1 million cells, S2—median: 30, IQR: 13–35 ASCs/1 million cells), compared to individuals who are ≥60 (S1—median: 3, IQR: 1–16.2 ASCs/1 million cells, S2—median: 1, IQR: 1–1 ASCs/1 million cells). Although the frequency of ASCs to the S1 protein significantly decreased with age (Spearman's *r* = −.57, *p* = .01), no such association was seen with responses to S2 and the N protein (Figure 2C).

Further, the frequency of ASCs was correlated with T cell responses to S1 and S2 separately. There was no significant correlation between ASCs and T cell responses to both S1 peptide pool (*p* = .66) and S2 peptide pool (*p* = .19). However, positive response to both T cell and memory B cells were shown by 14/20 individuals for S1 peptide pool and 15/20 individuals for S2 peptide pool.

### 3.6 Kinetics of antibody responses and T cell responses over time

**Figure 3:**
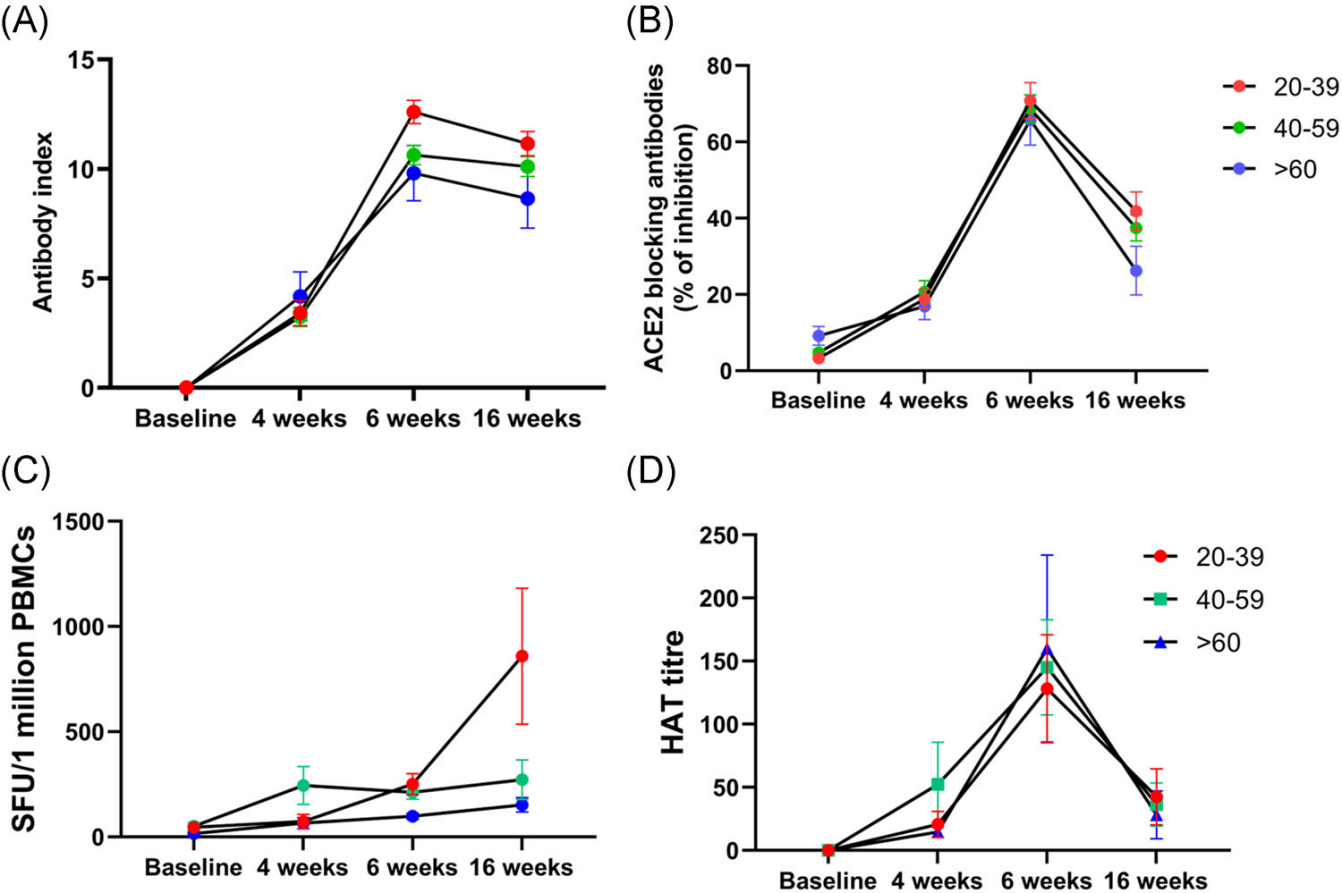
Kinetics of antibody and T cell responses over time. SARS‐CoV‐2 total antibodies were measured in 174 individuals, (49 in 20–39, 108 in the 40–59 and 17 in ≥60 years age group), at baseline, 4 weeks after the first dose, 6 weeks (2 weeks after the 2nd dose) and at 16 weeks (12 weeks post 2nd dose) after the second dose by ELISA. The decline in antibody responses from 6 to 16 weeks was not statistically significant in any age group (A). ACE2 receptor antibodies were measured by the surrogate virus neutralizing test in 92 individuals (32 in 20–39, 43 in 40–59, and 17 in the ≥60 years age group). The decline in antibody levels from 6 to 16 weeks was significant in 40–59 (*p* = .0007) and ≥60 (*p* = .005) age group (B). Ex vivo IFNγ ELISpot responses to the S protein overlapping pool of peptides were measured in 37 individuals, with 10 in the 20–39 age group, 18 in the 40–59 age group and 9 in the ≥60 age group. There was no difference (*p* = .8) in responses between 6 and 16 weeks (C). Antibodies to the RBD of the WT was measured by the haemagglutination assay test in 92 individuals (32 in 20–39, 43 in 40–59 and 17 in the ≥60 years age group). The decline in antibody levels from 6 to 16 weeks was significant in 20–39 (*p* < .0001), 40–59 (*p* < .0001) and ≥60 (*p* = .0002) age group (D). The lines indicate the mean, and the error bars indicate the standard error of the mean. All tests were two‐tailed. ELISA, enzyme‐linked immunosorbent assay; IFNγ, interferon gamma

Although initially we planned to follow the whole cohort that was initially recruited to the study, at the time of recruitment, we could only obtain blood samples from 174 individuals at all 4 time points (baseline, 4 weeks from first dose, 2 weeks from second dose and 3 months from second dose) to measure SARS‐CoV‐2 specific total antibodies. For this analysis, we were able to follow 49 individuals in the 20–39 age group, 108 in the 40–59 age group and 17 in ≥60 years age group. From the second dose, the SARS‐CoV‐2 total antibodies, measured by the Wantai antibody assay, declined in all the age groups but this decline was not significant in any of the age groups by the Friedman test (Figure 3A). In the 20–39, there was a 1.12‐fold reduction in the total antibodies from 6 to 16 weeks, whereas in 40–59 age group there was no reduction. In the ≥60 age group, there was a 1.13‐fold reduction in the total antibodies from weeks 6 to 16.

We could only recruit 92 individuals to measure the variability of ACE2 receptor blocking antibodies over time. These assays were carried out in 32 individuals in the 20–39 age group, 43 in the 40–59 age group and 17 in the ≥60 years age group. We saw a decline in ACE2 receptor blocking antibodies in all age groups from 6 to 16 weeks postfirst dose, which was more marked in the ≥60 years old age group (Figure 3B). The decline in antibody levels were significant among the 40–59 (*p* = .0007) and ≥60 (*p* = .005) age groups. There was no significant difference in ACE2 receptor blocking antibodies for 4 weeks after the first dose, compared to 3 months after the second dose in 20–39 years old (*p* = .09), 40–59 years old (*p* = .22) and ≥60 years old (*p* > .99). In the 20–39 age group from 6 to 16 weeks postfirst dose, there was a 1.7‐fold reduction in the levels of ACE2 receptor blocking antibodies (% of inhibition), while in the 40–59 age group there was a 1.8‐fold reduction and a 2.5‐fold reduction in the ≥60 years age group.

To explore the kinetics of ex vivo IFNγ ELISpot responses over time, we only had all four time points in 37 individuals, with 10 in the 20–39 age group, 18 in the 40–59 age group and 9 in the ≥60 age group. In contrast to observations with the antibody responses, we saw the frequency of responses to the overlapping peptides of the spike protein increase over time in those in the 20 to 39 age group, while the responses in the 40–59 and ≥60 age group remained unchanged (Figure 3C). However, the increase in the ex vivo IFNγ responses for both S1 and S2 in the 20–39 group from 6 weeks to 3 months (16 weeks), was not significant (*p* > .99).

We also investigated the change in the antibody titres to the RBD of the WT in 92 individuals (32 in 20–39, 43 in 40–59 and 17 in the ≥60 years age group) by the haemagglutination assay. The decline in antibody levels from 6 to 16 weeks was significant in 20–39 (*p* < .0001), 40–59 (*p* < .0001) and ≥60 (*p* = .0002) age group (Figure 3D). The HAT titres were only significantly higher at 16 weeks values than the 4‐week values (4 weeks after a single dose) in the 20–39 age group (*p* = .003), whereas the HAT titre to the RBD of the WT was similar the responses seen at 4 weeks in the 40–59 (*p* > .99) and ≥60 age group (*p* > .99).

## 4 DISCUSSION

In this study, we have determined the antibody and T cell responses in a cohort of Sinopharm/BBIBP‐CorV vaccinated individuals that we have been following throughout for 16 weeks. Three months (12 weeks) following the second dose we found that 95.07% of individuals had detectable SARS‐CoV‐2 specific total antibodies, although the antibody levels significantly declined with age. Although we did not measure neutralizing antibodies, we used the sVNT to measure ACE2 receptor blocking antibodies, which have shown to correlate with neutralizing antibodies.^17^ Based on this assay, 60.09% of individuals had ACE2 receptor blocking antibodies, although only 38.1% of those ≥60 years of age had detectable blocking antibodies. These ACE2 receptor blocking antibodies had declined in all individuals within a period of 16 weeks from receiving the first dose. Significant reductions from 2 weeks from the second dose was seen in individuals >40 years of age. Although the clinical implications of the decline in these antibody responses are not known, neutralizing antibodies have shown to correlate with protection from infection.^24^ Although neutralizing antibodies also are shown to correlate with prevention of symptomatic infection,^25^ it is yet unclear if it prevents severe illness.

Using the HAT assay, we measured antibodies to the RBD of the WT and VOCs in these different age groups. A total of 14.3%–16.7% individuals in the 20–39 age groups had detectable antibodies by this assay, while the positivity rates of those ≥60 years of age was <10%. Interestingly, significant differences were not seen between positivity rates to WT versus VOCs in these individuals, although the mean HAT titres were lower in all individuals to B.1.351 compared to other VOCs. The HAT assay was also shown to correlate with neutralizing antibodies.^26^ Therefore, based on the HAT assay and the sVNT assay, the presence of neutralizing antibodies levels appears to be low or undetectable in all age groups, but especially in those ≥60 years of age. At 12 weeks from the second dose a 1.7–2.5 reduction of ACE2 receptor blocking antibodies were seen, with significant reductions in HAT titres to the RBD of the WT in all age groups. It was shown that the BNT162b2 (Pfizer–BioNTech) and AZD1222 too had a twofold and fivefold reduction of antibodies to the S‐protein respectively, 70 days following the second dose of the vaccine.^5^ Our data showed that the ACE2 receptor blocking antibody levels were detectable in 75.9% individuals at >16 weeks after a single dose of AZD1222,^27^ whereas only 60.09% of individuals who received the two doses of Sinopharm/BBIBP‐CorV vaccine were positive for these antibodies at 12 weeks following the second dose. Therefore, although a significant decline has also been observed with BNT162b2 (Pfizer–BioNTech) and AZD1222 with time, it would be important to compare the decline in antibody responses in different vaccines with time. However, as it is unclear if the reduction in neutralizing antibodies, we have detected in the circulation would result in increased susceptibility to severe disease. Long‐ term efficacy studies are urgently needed to determine if such a reduction in circulating antibodies, while B and T cell memory is maintained, will result in enhanced risk of severe outcomes from infection.

Memory B cell responses have shown to be more durable and have shown to provide long lasting immunity^10^ and have shown to increase with time following natural infection.^28^ We found that 40%–45% of individuals had SARS‐CoV‐2 specific ASCs, 12 weeks following the second dose of the vaccine, while 28.6% of individuals had detectable ex vivo T cell responses. Although the SARS‐CoV‐2 specific antibody responses had declined with time, the frequency of ex vivo IFNγ ELISpot responses increased in the 20–39 age group from 2 weeks following the second dose to 12 weeks, while it remained unchanged in those in the 40–59 and ≥60 age group. Studies have shown that BNT162b2 and mRNA‐1273 induced persistence T cell responses even up to 8 months after vaccination^29^ and for AZD1222. Spike protein specific follicular helper T cells were also shown to persist in lymph nodes, 6 months following mRNA vaccines.^30^ However, the presence of a SARS‐CoV‐2 specific functional T cell response was found to be impaired in older individuals.^31^ For the Sinopharm/BBIBP‐CorV vaccine, we found that 43.6% of those between 20 and 39 years of age gave a positive response compared to 14.3% of those between 40 and 59 years of age and 33.3% ≤60 years old. Although this was not significant, younger individuals (20–39 years old), were more likely to have functional ex vivo T cell responses compared to older individuals. However, the overall T cell responses generated following the Sinopharm/BBIBP‐CorV vaccine (28.6%) was lower than those observed following the BNT162b2 vaccine (73%–74%).^29^, ^31^ Early appearance of T cell responses have been shown to associate with reduced clinical disease severity.^32^, ^33^ Therefore, although antibody responses declined over time following the Sinopharm/BBIBP‐CorV vaccine, the presence of a sustained memory B cell and a T cell response, could prevent the occurrence of severe illness, although breakthrough infection might still occur. Due to the limited number of B cell ELISpots carried out, we did not have sufficient number of individuals to compare the variation of ASCs over time.

In summary, we have described the immune responses to the Sinopharm/BBIBP‐CorV vaccine, 12 weeks following the second dose of the vaccine. We show that while the SARS‐CoV‐2 specific total antibodies, and especially ACE2 receptor blocking antibodies and antibodies to the RBD significantly decline, the memory T cell and B cell responses persisted. Since the ACE2 receptor blocking antibodies was shown to significantly decline in all age groups and especially in the elderly, it is important to carry out long term efficacy studies to assess the waning of immunity on hospitalization and severe disease to decide on booster doses in different populations.

## AUTHOR CONTRIBUTIONS

*Conceptualization*: Chandima Jeewandara, Graham S. Ogg, Alain Townsend, Gathsaurie Neelika Malavige. *Methodology*: Chandima Jeewandara, Inoka Sepali Aberathna, Achala Kamaladasa, Pradeep Darshana Pushpakumara, Dinuka Guruge, Ayesha Wijesinghe. *Formal analysis*: Gayasha Somathilake, Pradeep Darshana Pushpakumara, Shyrar Tanussiya, Gathsaurie Neelika Malavige. *Investigation*: Achala Kamaladasa, Pradeep Darshana Pushpakumara, Banuri Gunasekera, Deshni Jayathilaka, Saubhagya Danasekara, Shyrar Tanussiya, Inoka Sepali Aberathna, TPJ, Ayesha Wijesinghe, TP, Heshan Kuruppu, Thushali Ranasinghe, Madushika Dissanayake, Michael Harvie, Anushika Mudunkotuwa, TM. *Data curation*: Inoka Sepali Aberathna, Thushali Ranasinghe, Gayasha Somathilake, Heshan Kuruppu, Osanda Dissanayake, Nayanathara Gamalath, Dinithi Ekanayake, Jeewantha Jayamali, TM. *Project administration*: Chandima Jeewandara, Dinuka Guruge, Ruwan Wijayamuni, Gathsaurie Neelika Malavige. *Funding acquisition*: Chandima Jeewandara, Gathsaurie Neelika Malavige, Graham S. Ogg, Alain Townsend, Tao Dong, Tiong K. Tan. *Writing original draft*: Gathsaurie Neelika Malavige, Graham S. Ogg. *Writing‐review and editing*: Gathsaurie Neelika Malavige, Graham S. Ogg, Alain Townsend, Tao Dong. *Validation*: Lisa Schimanski, Tiong K. Tan, Pramila Rijal.

## CONFLICTS OF INTEREST

The authors declare no conflicts of interest.

## ETHICS STATEMENT

Ethical approval was received by the Ethics Review Committee of Faculty of Medical Sciences, University of Sri Jayewardenepura. Informed written consent was obtained from patients.

## Data Availability

All data is available in the manuscript and the figures.
